# PGT-A Chromosomal Outcomes in Trophectoderm Biopsies from Women with Endometriosis: A Retrospective IVF Cohort Study

**DOI:** 10.3390/diagnostics16182946

**Published:** 2026-09-11

**Authors:** Luana Ghilea (Seleș), Viorela Romina Murvai, Goman Marius, Petronela Naghi, Laura Maghiar, Alin Bodog, Anca Carmen Huniadi

**Affiliations:** 1 Department of Surgical Sciences, Obstetrics and Gynecology, Faculty of Medicine and Pharmacy, University of Oradea, 410087 Oradea, Romania; alin.bodog@didactic.uoradea.ro (A.B.);; 2 Doctoral School of Biological and Biomedical Sciences, University of Oradea, 1 University Street, 410087 Oradea, Romania; 3 Department of Obstetrics and Gynecology, Calla—Infertility Diagnostic and Treatment Center, Constantin A. Rosetti Street, 410103 Oradea, Romania; dr.rominamurvai@gmail.com (V.R.M.); marius_goman28@yahoo.com (G.M.); 4 Department of Embryology, Calla—Infertility Diagnostic and Treatment Center, Constantin A. Rosetti Street, 410103 Oradea, Romania; petronelanaghi@gmail.com

**Keywords:** endometriosis, IVF, PGT-A, mosaic results, trophectoderm biopsy, aneuploidy, reproductive genetics, assisted reproduction

## Abstract

**Background/Objectives:** Endometriosis is frequently associated with infertility and may affect several aspects of reproductive competence. However, whether women with endometriosis exhibit different preimplantation genetic testing for aneuploidy (PGT-A) outcomes compared with women without endometriosis remains uncertain. This study aimed to compare the distribution of PGT-A chromosomal outcomes—including euploid, mosaic, aneuploid, and complex aneuploid results—between women with and without endometriosis undergoing IVF. **Methods:** This retrospective observational study included 160 women who underwent IVF treatment between January 2023 and January 2026, including 55 women with endometriosis and 105 controls without evidence of endometriosis. A total of 129 blastocysts underwent trophectoderm biopsy and PGT-A, of which 120 yielded conclusive chromosomal results. PGT-A outcomes were classified as euploid, mosaic, aneuploid, or complex aneuploid. The overall distribution of the four PGT-A outcome categories was compared between groups using an unadjusted chi-square test. **Results:** Among blastocysts with conclusive PGT-A results, the proportions of euploid, mosaic, aneuploid, and complex aneuploid embryos were 31.6%, 18.4%, 26.3%, and 23.7%, respectively, in the endometriosis group and 42.7%, 14.6%, 25.6%, and 17.1%, respectively, in the control group. The overall distribution of PGT-A chromosomal outcome categories did not differ significantly between groups (χ^2^ = 1.65, df = 3, *p* = 0.648). **Conclusions:** In this retrospective cohort, no statistically significant difference was observed in the overall distribution of PGT-A chromosomal outcomes between women with endometriosis and controls. Although numerical differences were observed across individual outcome categories, the absence of statistical significance should not be interpreted as evidence of equivalence between the groups, particularly given the limited precision of the estimates. Larger patient-level studies accounting for within-patient clustering and relevant clinical and embryological confounders are needed to further clarify the relationship between endometriosis and PGT-A outcomes.

## 1. Introduction

Endometriosis is a chronic, estrogen-dependent inflammatory disorder characterized by the presence of endometrial-like tissue outside the uterine cavity and affects approximately 10% of women of reproductive age worldwide [1,2]. The disease is frequently associated with chronic pelvic pain, dysmenorrhea, dyspareunia, and infertility and represents an important cause of impaired reproductive health [2,3]. Although the association between endometriosis and infertility has been extensively investigated, the mechanisms through which the disease may influence reproductive outcomes remain incompletely understood.

Several mechanisms have been proposed to contribute to endometriosis-associated infertility, including altered folliculogenesis, chronic inflammation, oxidative stress, immune dysregulation, impaired oocyte competence, and altered endometrial receptivity [3,4,5]. In particular, the inflammatory and oxidative microenvironment associated with endometriosis may affect follicular development and oocyte quality through altered cytokine signaling, reactive oxygen species, and other inflammatory mediators [4,5,6]. These alterations may potentially influence fertilization and embryo development, although their clinical impact remains heterogeneous among affected women.

Assisted reproductive technologies (ART), particularly in vitro fertilization (IVF), provide an opportunity to investigate reproductive outcomes in women with endometriosis at several stages of the reproductive process. Studies evaluating IVF outcomes in this population have produced inconsistent findings. Some have reported impaired ovarian response, reduced oocyte quality, altered embryo development, or lower implantation and pregnancy rates, whereas others have observed comparable outcomes after accounting for maternal age, ovarian reserve, and other clinical factors [7,8,9]. These discrepancies suggest that the reproductive consequences of endometriosis are heterogeneous and may not be adequately characterized by conventional embryological outcomes alone.

The increasing clinical use of preimplantation genetic testing for aneuploidy (PGT-A) has enabled the assessment of chromosomal findings in trophectoderm biopsy samples obtained from blastocyst-stage embryos [10,11]. In addition to the conventional distinction between euploid and aneuploid results, contemporary PGT-A may identify mosaic and more complex chromosomal abnormalities, providing a broader description of the chromosomal patterns detected in trophectoderm samples [10,11,12]. Importantly, PGT-A findings derived from trophectoderm biopsy represent the chromosomal constitution of the sampled cells and should not be considered a direct assessment of the entire embryo, particularly in the context of mosaicism [12,13,14].

Advances in genetic testing have revealed a heterogeneous spectrum of PGT-A findings beyond the conventional euploid/aneuploid distinction [10,11,12]. Mosaic results represent a particularly complex biological and clinical category because abnormal and apparently normal cell populations may coexist within the sampled trophectoderm, while their distribution across the blastocyst may not be uniform [12,13,14]. Nevertheless, selected embryos classified as mosaic by PGT-A may retain reproductive potential and have resulted in healthy live births [15,16,17]. These observations support the evaluation of PGT-A findings across multiple chromosomal categories rather than relying exclusively on a binary euploid/aneuploid classification.

Mosaic chromosomal patterns are generally considered to arise predominantly from post-zygotic mitotic segregation errors occurring during early embryonic development [12,18]. Such events can generate genetically heterogeneous cell populations and contribute to the spectrum of chromosomal findings detected during PGT-A [12,18]. However, classification may also be influenced by the proportion and distribution of abnormal cells, trophectoderm sampling, the analytical platform, and laboratory-specific thresholds [13,14,19]. Accordingly, mosaicism detected by PGT-A should be interpreted as a genetic testing outcome rather than as direct evidence of whole-embryo chromosomal instability.

The relationship between endometriosis and PGT-A chromosomal outcomes remains incompletely characterized. Several studies have reported comparable aneuploidy or euploidy rates in women with endometriosis and appropriately matched controls [20,21,22]. Juneau et al. found aneuploidy rates comparable with those of age-matched IVF patients [20], while subsequent studies by Vaiarelli et al. and Bishop et al. similarly failed to demonstrate a detrimental effect of endometriosis on euploid blastocyst rates or reproductive outcomes following transfer of euploid embryos [21,22]. Other studies, however, have reported differences in ovarian response, oocyte characteristics, embryo development, or other IVF outcomes [7,8,9,23,24]. Together, these findings suggest that endometriosis may influence reproductive competence through mechanisms that are not necessarily reflected by a generalized increase in embryonic aneuploidy.

Maternal age is an established determinant of embryonic chromosomal abnormalities and the probability of obtaining euploid blastocysts [25]. A previous analysis from the same center and underlying cohort examined developmental and chromosomal competence across successive stages of IVF treatment [26]. The present study represents a secondary analysis focused specifically on the distribution of PGT-A chromosomal findings among biopsied blastocysts. Rather than limiting the assessment to overall euploidy, we examined euploid, mosaic, aneuploid, and complex aneuploid results as separate outcome categories. This analysis was intended to provide a more detailed descriptive characterization of PGT-A findings rather than to propose a novel PGT-A classification system.

Accordingly, the aim of the present study was to compare the distribution of euploid, mosaic, aneuploid, and complex aneuploid PGT-A results between women with endometriosis and controls without evidence of endometriosis undergoing IVF treatment. Given the exploratory nature of the study, we assessed whether the distribution of PGT-A findings differed between groups without assuming an independent detrimental effect of endometriosis on embryonic chromosomal competence.

## 2. Materials and Methods

### 2.1. Study Design and Population

This retrospective observational cohort study was conducted using clinical, embryological, and genetic data from women who underwent in vitro fertilization (IVF) treatment at the Calla IVF Center, Oradea, Romania, between January 2023 and January 2026. The study was designed to compare preimplantation genetic testing for aneuploidy (PGT-A) chromosomal outcomes between women with endometriosis and women without evidence of endometriosis.

Ethical approval for the retrospective analysis of anonymized clinical, embryological, and genetic data was obtained from the Institutional Review Board of the Calla IVF Center (Approval No. 3678/A, 5 January 2026). The study was conducted in accordance with the principles of the Declaration of Helsinki and its subsequent amendments.

A total of 160 women were included in the study, comprising 55 women diagnosed with endometriosis and 105 women without evidence of endometriosis who served as the control group. Eligibility for the overall cohort required complete clinical and embryological records. For the PGT-A analysis, only women with at least one blastocyst undergoing PGT-A and with an available genetic result were included in the chromosomal analytical population. Each woman contributed embryos from a single IVF/ICSI cycle, and no patient contributed blastocysts from more than one treatment cycle. In the endometriosis group, one blastocyst was biopsied per woman, whereas in the control group individual women contributed between one and three biopsied blastocysts.

The present study represents a secondary analysis of the cohort previously reported by Ghilea Seleș et al. [26]. The two studies share the same underlying population of 160 women (55 with endometriosis and 105 controls) treated at the same center between January 2023 and January 2026. The previous study evaluated sequential developmental outcomes across IVF stages and included an initial assessment of chromosomal competence, whereas the present analysis specifically focuses on the detailed distribution of PGT-A findings among biopsied blastocysts, including euploid, mosaic, aneuploid, and complex aneuploid results. Accordingly, the present study should be considered a secondary analysis addressing a distinct PGT-A-focused research question rather than an independent cohort. Although both studies derive from the same underlying cohort of 160 women, the PGT-A analytical populations differ because the present PGT-A-focused study involved a comprehensive re-evaluation of the original clinical, embryological, and genetic records and included all women meeting the PGT-A eligibility criteria defined for the current analysis. Consequently, the present analytical population comprised 123 women contributing 129 biopsied blastocysts, whereas the previous study [26] reported a more limited PGT-A subgroup of 18 patients contributing 41 biopsied blastocysts.

Patients were excluded if relevant clinical, embryological, or genetic records were incomplete; the IVF cycle was canceled before embryo biopsy; PGT-A results were unavailable; donor oocytes were used; or severe uterine abnormalities were present that could affect reproductive outcomes.

Because this was a retrospective study based on routine clinical practice, PGT-A was not performed for research purposes. Only PGT-A results generated as part of the patients’ clinical management were included in the analysis.

### 2.2. Diagnosis of Endometriosis

Women were assigned to the endometriosis group based on a previously established diagnosis, supported by clinical history, transvaginal ultrasound findings, magnetic resonance imaging when available, previous laparoscopic findings, and/or histopathological confirmation, as documented in the patients’ medical files.

Women in the control group underwent IVF/ICSI for infertility indications unrelated to endometriosis and had no clinical, imaging, or previously documented surgical evidence of endometriosis. Thus, the control group represented women without evidence of endometriosis rather than healthy fertile controls.

Detailed revised American Society for Reproductive Medicine (rASRM) staging was not consistently available in the retrospective records and was therefore not included in the comparative analyses. Consequently, the present study was not designed to evaluate associations between endometriosis stage and PGT-A outcomes.

### 2.3. Controlled Ovarian Stimulation

Controlled ovarian stimulation was performed using individualized protocols, predominantly based on a gonadotropin-releasing hormone (GnRH) antagonist regimen, according to routine clinical practice. Gonadotropin treatment included recombinant follicle-stimulating hormone (FSH; Gonal-F, Puregon, Rekovelle, or Bemfola) and/or human menopausal gonadotropin (hMG; Menopur or Pergoveris), with doses individualized according to maternal age, ovarian reserve parameters, and previous ovarian response.

Final oocyte maturation was triggered using either human chorionic gonadotropin (hCG) or a GnRH agonist according to the patient’s clinical characteristics and risk of ovarian hyperstimulation syndrome. Transvaginal ultrasound-guided oocyte retrieval was performed 34–36 h after triggering.

### 2.4. Fertilization and Embryo Culture

Fertilization was achieved by conventional in vitro fertilization (IVF) or intracytoplasmic sperm injection (ICSI), according to semen characteristics and the clinical indication. Following fertilization, embryos were cultured to the blastocyst stage using GTL culture medium (Vitrolife, Gothenburg, Sweden) in tri-gas incubators under standardized laboratory conditions. Incubation was maintained at 37 °C, with approximately 5.5% CO_2_ and 5% O_2_, and culture media were pre-equilibrated before use to minimize temperature and pH fluctuations.

Blastocyst morphology was assessed by experienced embryologists according to the Gardner and Schoolcraft grading system, considering the degree of blastocyst expansion together with inner cell mass (ICM) and trophectoderm (TE) morphology. Blastocyst expansion was graded on a scale from 1 to 6, while ICM and TE morphology were classified as A, B, or C according to morphological quality.

Blastocysts considered suitable for PGT-A underwent trophectoderm biopsy on Day 5, Day 6, or Day 7, according to developmental progression and morphological suitability.

### 2.5. Trophectoderm Biopsy and Preimplantation Genetic Testing for Aneuploidy

Trophectoderm biopsy was performed on expanded blastocysts selected for PGT-A. A laser-assisted opening of the zona pellucida was created using a Hamilton Thorne LYKOS^®^ laser system (Hamilton Thorne, Inc., Beverly, MA, USA), after which approximately 4–5 trophectoderm cells were removed using a biopsy pipette. The biopsied cells were transferred into PCR tubes containing approximately 2 µL of buffer, stored at −20 °C, and subsequently processed for genetic analysis. Following biopsy, blastocysts were vitrified within approximately 90 min using the Kitazato vitrification protocol.

PGT-A was performed using next-generation sequencing (NGS) with the Ion ReproSeq™ PGS Kit (Thermo Fisher Scientific, Waltham, MA, USA), according to the validated laboratory workflow used during the study period. Chromosomal copy-number profiles were evaluated across all chromosomes. As this was a retrospective study, the final classifications reported by the genetic laboratory were used for the present analysis.

For the four-category analysis, each conclusive PGT-A result was assigned to one of the mutually exclusive outcome categories according to the final laboratory-reported classification: euploid, mosaic, aneuploid, or complex aneuploid. Euploid results showed no reported chromosomal copy-number abnormality. Mosaic results were those reported as mosaic based on an intermediate copy-number signal. Aneuploid results comprised non-mosaic abnormal profiles involving a single chromosomal abnormality, whereas complex aneuploid results comprised non-mosaic abnormal profiles involving multiple chromosomal abnormalities. Thus, each conclusive trophectoderm biopsy was counted once and only once in the four-category comparative analysis. The revised mutually exclusive definitions did not result in reclassification of any biopsy, as the original laboratory reports already distinguished non-mosaic single-chromosome abnormalities from non-mosaic multiple-chromosome abnormalities.

Mosaic classification was based on an intermediate chromosomal copy-number signal within the genetic laboratory’s reporting range of 20–80%. The reported mosaic estimate therefore represents an inferred contribution of abnormal cells derived from the copy-number signal of the pooled multicellular trophectoderm biopsy and not a direct count of abnormal cells.

Biopsies yielding failed, inconclusive, or non-informative PGT-A results were not assigned to any of the four chromosomal outcome categories and were excluded from the comparative analysis of conclusive results; these outcomes were reported separately.

Because PGT-A was performed on multicellular trophectoderm biopsy samples, the reported chromosomal classifications, particularly mosaic findings, reflect the copy-number profile of the sampled trophectoderm tissue. They should therefore not be interpreted as direct evidence of the chromosomal constitution of the entire embryo or of the inner cell mass.

### 2.6. Study Outcomes

The primary outcome was the overall distribution of conclusive PGT-A chromosomal results euploid, mosaic, aneuploid, and complex aneuploid between women with endometriosis and controls without evidence of endometriosis.

Secondary outcomes included the total number of blastocysts undergoing trophectoderm biopsy, the number of blastocysts yielding conclusive PGT-A results, and the relative proportions of each PGT-A chromosomal category within the two study groups. The study was designed to characterize and compare PGT-A outcome distributions and not to infer the chromosomal constitution or genetic competence of the entire embryo from trophectoderm biopsy findings.

### 2.7. Statistical Analysis

Statistical analyses were performed using IBM SPSS Statistics version 26.0 (IBM Corp., Armonk, NY, USA). Continuous variables were summarized as mean ± standard deviation (SD), whereas categorical variables were presented as absolute frequencies and percentages. Baseline clinical and embryological characteristics were summarized descriptively for the overall study cohort.

The distribution of conclusive PGT-A outcomes across the four predefined chromosomal categories—euploid, mosaic, aneuploid, and complex aneuploid—was compared between the endometriosis and control groups using an unadjusted chi-square test. This analysis was considered a secondary descriptive comparison performed at the blastocyst level. Although each woman contributed embryos from a single IVF/ICSI cycle, more than one biopsied blastocyst could originate from the same woman in the control group. Therefore, blastocyst-level observations could not be considered fully independent. Given the observed distribution of biopsied blastocysts, within-patient clustering was minimal: each woman in the endometriosis group contributed one biopsied blastocyst, while women in the control group contributed between one and three blastocysts, corresponding to an average of approximately 1.07 blastocysts per woman. Therefore, although complete independence of blastocyst-level observations cannot be assumed, the degree of clustering was considered negligible, and the unadjusted chi-square test was retained as the primary descriptive analysis.

All statistical tests were two-sided, and a *p*-value < 0.05 was considered statistically significant.

## 3. Results

### 3.1. Baseline Characteristics of the Study Population

A total of 160 women undergoing IVF treatment were included in the study, comprising 55 women with endometriosis and 105 controls without evidence of endometriosis. Baseline demographic and embryological characteristics of the overall study cohort are summarized in Table 1.

The mean maternal age of the overall cohort was 36.06 ± 4.20 years. The mean number of retrieved oocytes was 8.95 ± 7.91, of which 6.11 ± 5.03 were mature (MII) oocytes. The mean number of fertilized oocytes was 4.74 ± 3.90, while the mean number of embryos obtained was 2.27 ± 2.11. These descriptive characteristics provide the clinical and embryological context for the subsequent analysis of PGT-A chromosomal outcomes in women with and without endometriosis.

### 3.2. Genetic Outcomes Following PGT-A

**Figure 1:**
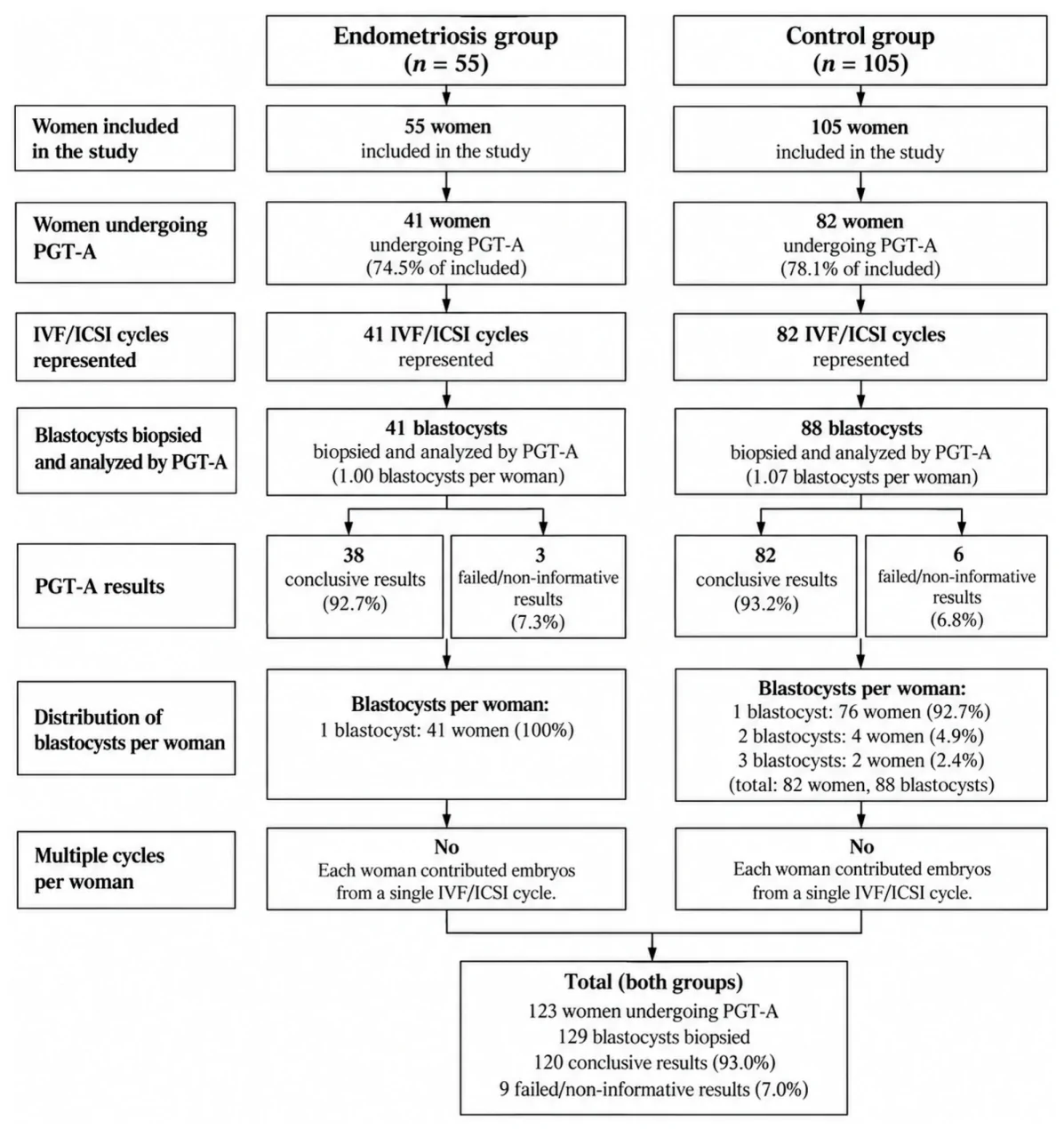
Flow diagram of the study population and PGT-A analytical cohort.

Preimplantation genetic testing for aneuploidy (PGT-A) was performed on blastocyst-stage embryos from both study groups. The flow of the study population from the overall IVF cohort to the PGT-A analytical population is presented in Figure 1. A total of 129 blastocysts underwent trophectoderm biopsy and PGT-A, including 41 blastocysts from women with endometriosis and 88 from controls. Conclusive chromosomal results were obtained for 120 blastocysts, comprising 38 in the endometriosis group and 82 in the control group. Nine biopsied blastocysts yielded failed, inconclusive, or non-informative results, including 3 in the endometriosis group and 6 in the control group, and were excluded from the four-category comparative analysis.

The diagram presents, separately for women with endometriosis and controls, the numbers of women included in the overall cohort, women undergoing PGT-A, IVF/ICSI cycles, blastocysts biopsied, conclusive PGT-A results, failed/inconclusive/non-informative results, and the distribution of biopsied blastocysts per woman.

Among blastocysts with conclusive PGT-A results, 12/38 (31.6%) were classified as euploid, 7/38 (18.4%) as mosaic, 10/38 (26.3%) as aneuploid, and 9/38 (23.7%) as complex aneuploid in the endometriosis group. In the control group, the corresponding distributions were 35/82 (42.7%), 12/82 (14.6%), 21/82 (25.6%), and 14/82 (17.1%), respectively (Table 2).

Numerically, the endometriosis group showed a lower proportion of euploid results (31.6% vs. 42.7%). Higher proportions of mosaic (18.4% vs. 14.6%) and complex aneuploid results (23.7% vs. 17.1%) were observed compared with controls. The proportions of aneuploid results were similar between groups (26.3% vs. 25.6%). However, the overall distribution across the four PGT-A outcome categories did not differ significantly between the endometriosis and control groups (χ^2^ = 1.65, df = 3, *p* = 0.648).

Accordingly, the observed numerical differences in individual PGT-A categories were not statistically significant at the level of the overall distribution and should be regarded as descriptive and exploratory rather than as evidence of an endometriosis-associated alteration in chromosomal outcomes. In addition, because the analysis was conducted at the blastocyst level and multiple blastocysts could originate from the same woman, these unadjusted comparisons should be interpreted with caution.

The largest absolute between-group difference was observed for euploid results, which were 11.1 percentage points lower in the endometriosis group than in controls (absolute difference: −11.1 percentage points; 95% CI: −29.4 to 7.1). The confidence intervals for all four between-group differences included zero and were relatively wide, indicating limited precision of the estimates.

## 4. Discussion

The present study evaluated PGT-A chromosomal outcomes in women with endometriosis undergoing IVF by examining the distribution of euploid, mosaic, aneuploid, and complex aneuploid results. The principal finding was that the overall distribution of these chromosomal outcome categories did not differ significantly between women with endometriosis and controls without evidence of endometriosis (χ^2^ = 1.65, *p* = 0.648). Although numerical differences were observed across individual categories, particularly for euploid, mosaic, and complex aneuploid results, they were not statistically significant. Accordingly, the findings should be interpreted as descriptive and exploratory and should not be considered evidence of equivalence between the groups or of an independent effect of endometriosis on PGT-A chromosomal outcomes.

Among blastocysts with conclusive PGT-A results, the proportion classified as euploid was numerically lower in women with endometriosis than in controls (31.6% vs. 42.7%). However, this difference must be interpreted with caution in light of the sample size and the lack of a statistically significant difference in the overall distribution of chromosomal outcomes. Importantly, previous studies have similarly failed to demonstrate a consistent detrimental effect of endometriosis on embryonic euploidy. Juneau et al. reported aneuploidy rates in women with endometriosis comparable with those of age-matched IVF patients [20], while Vaiarelli et al. found no significant effect of endometriosis on the euploid blastocyst rate [21]. Bishop et al. likewise reported similar aneuploidy rates and no adverse effect of endometriosis on live-birth outcomes following frozen transfer of euploid blastocysts [22]. Together, these findings suggest that the reproductive effects of endometriosis may not necessarily be mediated through a generalized increase in embryonic chromosomal abnormalities.

The proportions of conventionally aneuploid results were nearly identical between the two groups (26.3% in women with endometriosis vs. 25.6% in controls), whereas mosaic (18.4% vs. 14.6%) and complex aneuploid results (23.7% vs. 17.1%) were numerically more frequent in the endometriosis group. These differences were not statistically significant and therefore cannot establish an association between endometriosis and either mosaicism or complex chromosomal abnormalities. They should instead be considered hypothesis-generating observations that may inform future adequately powered studies. This distinction is particularly important because mosaic PGT-A results represent intermediate copy-number patterns inferred from a multicellular trophectoderm biopsy rather than direct measurement of the proportion of abnormal cells throughout the embryo [12,13,14,19].

The biological mechanisms associated with endometriosis nevertheless provide a rationale for investigating reproductive competence beyond conventional IVF outcomes. Chronic inflammation, oxidative stress, immune dysregulation, and alterations in the follicular microenvironment have been implicated in impaired oocyte competence and early embryonic development [3,4,5,6]. Such mechanisms may affect reproductive potential without necessarily producing detectable differences in overall embryonic euploidy. The present findings are therefore compatible with the possibility that endometriosis-related reproductive impairment may occur at developmental, cellular, inflammatory, or implantation-related levels rather than through a generalized alteration in chromosome number. However, these mechanisms were not directly investigated in the present study, and no causal relationship between the endometriotic environment and the observed PGT-A patterns can be inferred.

This interpretation is also consistent with our previous analysis of the same underlying clinical cohort, which examined developmental competence across successive stages of IVF treatment and included a more limited analysis of chromosomal competence [26]. The present study represents a secondary analysis focused specifically on the distribution of PGT-A chromosomal findings among biopsied blastocysts. The overlap between the two studies should therefore be considered when interpreting the current findings: the present analysis does not represent an independent patient cohort but extends the previous investigation by examining the distribution of euploid, mosaic, aneuploid, and complex aneuploid PGT-A results as separate outcome categories. Accordingly, the contribution of the present study lies in the more detailed characterization of PGT-A findings within this cohort rather than in the introduction of a novel chromosomal classification system.

The interpretation of mosaic PGT-A findings warrants particular caution. Mosaic-range results are generally attributed to intermediate chromosome copy-number signals and may reflect post-zygotic mitotic errors during early embryonic development [12,18]. However, the relationship between a trophectoderm mosaic-range result and the chromosomal constitution of the entire embryo is not straightforward. Technical characteristics of the testing platform, biopsy sampling, analytical thresholds, and the distribution of abnormal cells within the blastocyst may all influence classification [13,14,19]. Current recommendations therefore emphasize that a mosaic PGT-A result should be interpreted as a finding derived from the tested trophectoderm sample rather than as direct evidence of uniform whole-embryo mosaicism [14,19].

Clinical experience has further demonstrated that selected embryos reported as mosaic can retain reproductive potential. Healthy live births following transfer of embryos with mosaic PGT-A results have been documented [15,16,17], and contemporary guidance recognizes that mosaic results encompass heterogeneous chromosomal patterns with potentially different reproductive implications [14,19]. These observations reinforce the need for cautious interpretation of mosaic findings and support reporting chromosomal outcomes beyond a simple binary euploid/aneuploid framework. At the same time, the present study was not designed to assess the reproductive potential of individual mosaic subtypes, and its results should not be used to guide embryo-transfer prioritization.

Several strengths of the present study should be acknowledged. The analysis directly compared PGT-A findings between women with endometriosis and controls without evidence of endometriosis and considered four chromosomal outcome categories. Maternal age was similar between the study groups, reducing the likelihood that major between-group differences in age accounted for the observed descriptive patterns. In addition, the use of a standardized IVF laboratory workflow and NGS-based PGT-A provided a consistent analytical framework throughout the study period.

The study also has important limitations. First, its retrospective observational design introduces the possibility of selection bias and precludes causal inference. Second, the number of blastocysts with conclusive PGT-A results was limited, particularly in the endometriosis group. The study may therefore have been insufficiently powered to detect modest differences between groups, and the absence of statistical significance should not be interpreted as evidence of equivalence.

Third, detailed information regarding endometriosis phenotype, rASRM stage, lesion severity, localization, previous ovarian surgery, endometrioma characteristics, and other potentially relevant clinical variables was not consistently available in the retrospective records. Additional potential confounders, including ovarian reserve, BMI, infertility indication, male-factor characteristics, stimulation-related variables, and clinical indications for PGT-A, could not be comprehensively incorporated into the present analysis. Residual confounding therefore cannot be excluded.

A further limitation concerns the characterization of mosaic results. Mosaic findings were analyzed as a single category because sufficiently detailed information regarding mosaic level and chromosomal pattern was not consistently available across the study population. Consequently, potential differences between lower- and higher-range mosaic results, whole-chromosome and segmental abnormalities, and different numbers or combinations of chromosomes involved could not be evaluated. Moreover, the percentage associated with a mosaic-range PGT-A result is inferred from the chromosome copy-number signal obtained from a pooled multicellular trophectoderm biopsy and should not be interpreted as a direct measurement of the percentage of abnormal cells within the embryo [14,19].

An additional methodological limitation concerns the unit of analysis. Chromosomal outcomes were compared at the blastocyst level, although multiple blastocysts could originate from the same woman and therefore cannot be considered fully independent observations. The unadjusted chi-square comparison does not account for this within-patient clustering. These limitations reinforce the exploratory nature of the current statistical findings. Future studies should use patient-level analyses or statistical approaches capable of accounting for repeated embryos within individual women, such as generalized estimating equations or generalized linear mixed-effects models.

The nine biopsied blastocysts without conclusive PGT-A results also require consideration because exclusion of non-informative or failed analyses may introduce additional selection into the final chromosomal outcome distribution. Their distribution across study groups and the reasons for inconclusive testing should therefore be reported transparently and taken into account when interpreting the findings.

Future studies should include larger, prospectively characterized cohorts with detailed information on endometriosis phenotype and severity and relevant reproductive and clinical confounders. Patient-level analyses that account for the number of embryos tested, maternal age, ovarian reserve, and other determinants of IVF and PGT-A outcomes would yield more robust estimates of potential associations. Further characterization of mosaic findings according to mosaic range, whole-chromosome versus segmental abnormalities, and the number and type of chromosomes involved may also provide additional insight. Integration of PGT-A findings with inflammatory, oxidative, transcriptomic, epigenetic, or metabolomic markers could further clarify whether specific biological features of endometriosis are associated with embryo development or chromosomal findings.

Overall, the present study did not demonstrate a statistically significant difference in the distribution of euploid, mosaic, aneuploid, and complex aneuploid PGT-A results between women with endometriosis and controls without evidence of endometriosis. Although the endometriosis group showed a numerically lower proportion of euploid results and higher proportions of mosaic and complex aneuploid results, these differences remain exploratory and should not be interpreted as evidence of equivalence or of an independent effect of endometriosis on embryonic chromosomal competence. Larger studies using appropriately adjusted patient-level or clustered analyses are required to determine whether endometriosis is associated with specific patterns of PGT-A chromosomal outcomes.

## 5. Conclusions

This study did not identify a statistically significant difference in the overall distribution of PGT-A chromosomal outcomes between women with endometriosis and controls without evidence of endometriosis. Although the endometriosis group showed numerically lower proportions of euploid embryos and higher proportions of mosaic and complex aneuploid embryos, these differences were not statistically significant and should be considered exploratory rather than evidence of an independent effect of endometriosis on embryonic chromosomal outcomes.

The absence of a statistically significant difference should not be interpreted as evidence of equivalence between women with and without endometriosis, particularly given the limited precision of the estimates and the relatively small PGT-A analytical sample.

Overall, the present findings do not support a definitive association between endometriosis and altered distributions of PGT-A chromosomal outcomes. Larger studies incorporating detailed endometriosis phenotyping, relevant clinical confounders, and statistical approaches accounting for multiple embryos within individual patients are needed to clarify whether endometriosis is independently associated with specific PGT-A chromosomal patterns.

## Figures and Tables

**Table 1:** Baseline clinical and embryological characteristics of the study cohort.

Variable	Overall Cohort (*n* = 160), Mean ± SD
Age (years)	36.06 ± 4.20
Retrieved oocytes (*n*)	8.95 ± 7.91
Mature oocytes (MII) (*n*)	6.11 ± 5.03
Fertilized oocytes (*n*)	4.74 ± 3.90
Embryos (*n*)	2.27 ± 2.11

Data are presented as mean ± standard deviation (SD). These descriptive characteristics refer to the overall cohort of 160 women and were previously reported for the same underlying cohort [26].

**Table 2:** Distribution of chromosomal outcomes following PGT-A.

Category	Endometriosis *n* = 38, *n* (%) [95% CI]	Control *n* = 82, *n* (%) [95% CI]	Absolute Difference, Percentage Points [95% CI]
Euploid	12 (31.6) [19.1–47.5]	35 (42.7) [32.5–53.5]	−11.1 [−27.6 to 7.7]
Mosaic	7 (18.4) [9.2–33.4]	12 (14.6) [8.6–23.9]	3.8 [−9.2 to 20.0]
Aneuploid	10 (26.3) [15.0–42.0]	21 (25.6) [17.4–36.0]	0.7 [−14.7 to 18.4]
Complex aneuploid	9 (23.7) [13.0–39.2]	14 (17.1) [10.5–26.6]	6.6 [−7.7 to 23.5]

Overall comparison: χ^2^ = 1.65, df = 3, *p* = 0.648. Note: Percentages were calculated among blastocysts with conclusive PGT-A results within each study group. Data are presented as numbers (percentages) with 95% confidence intervals (CIs). Absolute differences were calculated as endometriosis minus control and are expressed in percentage points. Confidence intervals for proportions were calculated using the Wilson score method, whereas confidence intervals for absolute differences between proportions were calculated using the Newcombe method. The unadjusted overall comparison was performed at the blastocyst level as a descriptive secondary analysis. Within-patient clustering was limited because each woman in the endometriosis group contributed one biopsied blastocyst, whereas women in the control group contributed between one and three.

## Data Availability

The original contributions presented in this study are included in the article. Further inquiries can be directed to the corresponding authors.
